# The trace that is valuable: serum copper and copper to zinc ratio for survival prediction in younger patients with newly diagnosed acute myeloid leukaemia

**DOI:** 10.1186/s12885-022-10486-7

**Published:** 2023-01-05

**Authors:** Taotao Li, Liming Shi, Wei Wei, Jiancheng Xu, Qiuju Liu

**Affiliations:** 1 Department of Haematology, the First Hospital of Jilin University, Cancer Center, Changchun, Jilin, China; 2 Department of Haematology, Zhongda Hospital, Medical School of Southeast University, Nanjing, China; 3 Department of Laboratory Medicine, First Hospital of Jilin University, Changchun, China

**Keywords:** Acute myeloid leukaemia, Copper, Zinc, Long-term survival

## Abstract

**Purpose:**

No data on predicting the survival of AML patients based on the level of trace elements in the serum have been presented to date. The aims of this prospective cohort study were as follows: (i) to evaluate the serum Cu and Zn levels in people from Northeast China, (ii) to assess the association between the serum Cu level (SCL) and Cu to Zn ratio (SCZR) and clinical and nutrition data, and (iii) to investigate the predictive values of the SCL and SCZR in newly diagnosed de novo AML patients.

**Methods:**

A total of 105 newly diagnosed AML patients and 82 healthy controls were recruited. The serum Cu and Zn levels were determined by inductively coupled plasma spectrometry. The associations of SCL and SCZR with the survival of these AML patients were assessed by Cox proportional hazards models.

**Results:**

Both SCL and SCZR were positively related to the blast percentage of bone marrow and C-reactive protein, negatively related to albumin level and *CEBPA* double mutation and were significantly associated with worse overall survival and disease-free survival. Meanwhile, patients with higher SCL had worse CTCAE levels, and patients with higher SCZR showed less complete remission during the first course of induction chemotherapy. Moreover, higher SCZR was positively associated with ELN risk stratification, and was negatively associated with haemoglobin level and prognostic nutritional index (PNI).

**Conclusion:**

The SCL and SCZR are associated with long-term survival in patients with newly diagnosed AML undergoing intensive induction and may serve as important predictive biomarkers.

**Supplementary Information:**

The online version contains supplementary material available at 10.1186/s12885-022-10486-7.

## Introduction

Acute myeloid leukaemia (AML) is a rare haematologic malignancy but is the most common subtype of acute leukaemia in adults and has the shortest survival rate (5-year survival = 24%) [1].The age-adjusted incidence of AML is 4.3 per 100,000 annually, and the median age of diagnosis is 68 years in the United States. With the emergence of an ageing population, the incidence of acute myeloid leukaemia will further increase with poor prognosis in elderly individuals [2]. Accurate evaluation of prognosis is critical for the management of AML. Currently, according to ELN risk stratification, AML is classified into three prognostic risk groups: favourable, intermediate, and adverse groups [3]. These are based on both cytogenetics and molecular biomarkers that predict different responses to standard therapeutics and survival. Meanwhile, clinical factors, increased age, and poor performance status are both associated with lower rates of complete remission (CR) and decreased overall survival (OS) [4]. Multivariate model analyses suggest that other variables, such as platelet count, serum creatinine, or albumin, account for most of the increased risk of treatment-related mortality (TRM) seen in AML patients [5]. However, these factors have not been added to the current system that differentiates the prognostic subsets in AML patients. For a specific AML patient, it can be difficult to make an accurate evaluation of the prognosis. Therefore, it is of great clinical significance to explore the comprehensive and prognostic factors that reflect the systemic status of the host, and the biological behaviour of leukaemia cells may provide novel and effective therapeutic ideas for AML patients.

Trace elements play a key role in human metabolism. Zinc (Zn) and copper (Cu) are two essential trace elements that are important cofactors for many enzymes and play a critical role in maintaining the integrity and stability of DNA. As two transition metals, Cu and Zn are associated with a variety of biological reduction and oxidation (redox) processes. Increasing data have indicated the possible coincidence and association of these trace element disturbances in the pathogenesis of cancer by dysregulation of the redox enzyme activity [6, 7]. In B-cell chronic lymphocytic leukaemia(CLL), excess Cu has been considered to be a potent oxidant and has a potential role in carcinogenesis and prognostic value [8, 9]. Copper is an acute phase reactant that increases in response to infection, injury, and chronic inflammatory conditions. Zinc has been particularly linked to immune processes, inflammation, and the metabolism of oxidative radicals. Recently, a systematic review regarding the association between serum Zn levels and outcomes in hospitalized patients showed that Zn deficiency was associated with longer stay and even death risk, although there were some limitations in the studies [10]. Therefore, the levels of these trace elements are related to the state of the body itself and the prognosis of malignancy. The serum levels of Zn and Se were significantly decreased and Cu levels were significantly increased in the serum of patients with AML [11, 12], and one earlier study showed that the levels of these micronutrients might be helpful in the prediction of response to induction chemotherapy in patients treated for AML [13]. However, we do not know the predictive value of these trace elements for long-term survival in AML patients. Better than the corresponding serum Cu and serum Zn levels, one study indicated that the serum copper to zinc ratio (SCZR) can be an indicator of the extent and prognosis of malignancy [14].

In this prospective case–control study, we evaluated the association of serum copper level (SCL) and SCZR at diagnosis with survival among younger patients with de novo AML from northeast China.

## Methods

### Subjects

This is a hospital-based, prospective case–control study. From October 2017 to May 2019, 105 newly diagnosed AML patients (ages ≥ 18 years old) at the haematology department were enrolled in this study, and 82 healthy subjects from the medical examination centre were enrolled as controls. The age of the patients and controls varied from 18 to 77 years. The diagnosis of AML was established according to the World Health Organization (WHO) 2016 classification. All AML subjects of the study were subjected to complete history taking, clinical examination, and routine laboratory investigations, including complete blood counts and biochemical tests, in the first early morning, on an empty stomach, and before any treatment. Among 105 AML patients, 38 patients were excluded from the survival analysis as follows: patients who had received induction chemotherapy other than the standard 3 + 7 (*n* = 5), less than 2 cycles of consolidation treatment (*n* = 22), older than 65 years old (*n* = 8), and patients with secondary AML (*n* = 8). Finally, 62 younger de novo AML patients (18–65 years old) were included in our endpoint survival analysis.

### Clinical treatment and response criteria

Patients were treated with the standard “3 + 7” (daunorubicin/idarubicin + cytarabine) regimen for induction chemotherapy. Bone marrow aspiration was performed on days 14 and 28 after chemotherapy. Response assessment was conducted according to the International Working Group (IWG) criteria for AML [15]. The second reinduction regimen was the same as the induction regimen if the patients achieved an outcome over partial remission(PR) or was changed to a high-dose cytarabine-based regimen such as CLAG (cladribine, cytarabine, G-CSF) if not. Consolidation therapy was administered with at least 2 cycles of high-dose cytarabine at 1.5 ~ 3.0 g/m^2^ (6 doses) or HCT according to the ELN 2017 risk stratification and patient choice. Adverse events were graded according to Common Terminology Criteria for Adverse Events v4.0 (CTCAE), published May 28, 2009, by the National Cancer Institute.

### Determination of Cu and Zn levels in the serum

For trace element detection, 5 ml of fasting venous blood sample was collected and placed in a vacuum EDTA-K_2_ anticoagulant blood collection vessel. After centrifugation at 3000 rpm for 10 min, the red blood cell fraction and plasma were removed, and the serum was transferred to a deionized cryotube and stored in a refrigerator at -20 °C until element analysis. The serum concentrations of Cu and Zn were evaluated using inductively coupled plasma spectrometry (ICP–MS, PerkinElmer Life and Analytical Sciences, Inc., CT, USA). For male participants with levels less than 0.70 mg/L and more than 1.40 mg/L and for female participants with levels less than 0.80 mg/L and more than 1.50 mg/L, the cut-offs were used to categorize hypo- and hypercupremia, respectively. The normal range of serum Zn levels was 0.76 mg/L to 1.50 mg/L. SCZR was evaluated as an alternative biomarker for assessing clinical outcomes [16].

### Clinical data collection

The patients’ general information was collected as follows: sex, age, height, weight and laboratory data, including complete blood count, albumin (ALB) level, C-reactive protein(CRP), triglycerides, and cholesterol levels, which were measured by the Laboratory Department of First Hospital of Jilin University. Morphology, immunophenotyping, genetics, and molecular biology were tested and analysed by The Haematology Laboratory of the Cancer Center. The prognostic nutritional index (PNI) calculation formula was serum albumin value (g/L) + 5 × total number of peripheral blood lymphocytes (× 10^9^/L).

### Statistical analysis

SCZR was calculated as the serum Cu concentration divided by the serum Zn concentration. Analysis of data was performed with the statistical package for the social science computer program (SPSS Inc., version 22.0, Chicago, IL) [17]. Numerical data are expressed as the mean ± standard deviation and range. Qualitative data were expressed as frequencies and percentages. Fisher’s exact test was used to examine the relation between qualitative variables. For quantitative data (normally distributed), comparisons between two groups were performed using Student’s *t* test (*P* < 0.05; significant). Numerical data not conforming to a normal distribution were analysed using the Mann-Whiney U test. A receiver operating characteristic (ROC) curve was drawn, and the best cut-off value of SCZR was determined with the maximum value of the Youden index [sensitivity-(1-specificity)]. Associations between potential predictors and SCL and SCZR were evaluated by Cox proportional hazards models. The Kaplan–Meier method was used to draw the survival curve, and comparisons between the groups were performed using the log-rank test. A multivariate Cox regression model was used to analyse the relationship between SCL and SCZR and the prognosis of AML patients and to calculate the hazard ratios (HRs) and their 95% confidence intervals (95% CIs). A *P* < 0.05 was considered to be statistically significant. Comparison of data was performed for the levels obtained at diagnosis.

## Results

### Participants’ characteristics

**Fig. 1:**
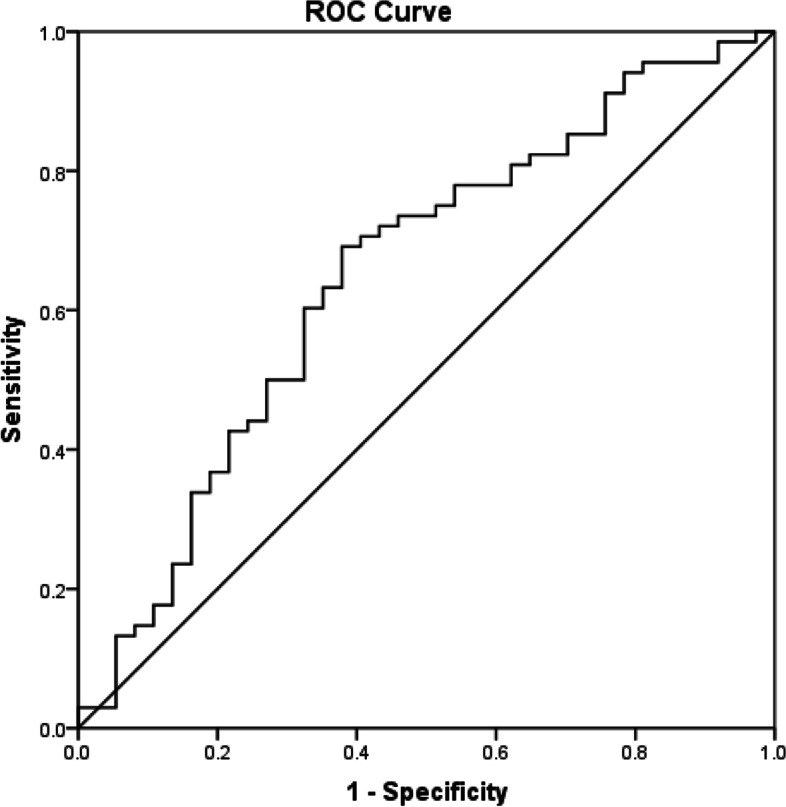
Receiver operating characteristics curves of serum copper to zinc ratio in patients with acute myeloid leukemia

As shown in Table 1, among 187 participants, 105 were newly diagnosed with adult AML, including 51 males and 54 females, with a median age of 50 years. A total of 82 patients were enrolled in the healthy control group, including 43 males and 39 females, with a median age of 44 years. There was no statistically significant difference in age or sex between the two groups. Sixty-two younger AML patients (shown in supplementary 1) who were treated with the standard “3 + 7” (*n* = 62) for induction chemotherapy and received at least 2 cycles of consolidation treatment were enrolled in the survival analysis. The ROC curve of the SCZR was plotted for 62 AML patients (Fig. 1). The area under the curve (AUC) was 0.650 (95% CI, 0.537–0.762), and the standard error was 0.057 (*p* = 0.011). The critical SCZR for the prognosis of AML was 1.56 (sensitivity, 69.1%; specificity, 62.2%). The median follow-up time of the 62 patients was 35.4 months (range: 0.25–47.46 months), and the median overall survival (OS) was 17 months.

**Table 1:** Comparison of serum copper, zinc, copper/zinc ratio level between AML patients and healthy controls

Variable	Total (*n* = 187)	AML patients (*n* = 105)	Controls (*n* = 82)	t/$\upchi$^2^/U	*p*-value
Sex,(M/F)	94/93	51/54	43/39	0.275	0.600
Age,(years)^&^	48.0(35.0–56.0)	50.0 (38.5–57.5)	44(32.8–56.0)	3599.0	0.054
Serum copper, (mg/L)^&^	1.056(0.93–1.27)	1.20(0.95–1.46)	1.01(0.91–1.10)	2753.0	< 0.001
Serum zinc,(mg/L)^*^	0.76 ± 0.16	0.68 ± 0.14	0.86 ± 0.10	-10.07	< 0.001
copper/zinc ratio^&^	1.36(1.11–1.86)	1.74(1.34–2.35)	1.21(1.06–1.34)	1546.0	< 0.001

Values are expressed as means *(standard deviations) or medians &(IQR interquartile ranges). $\upchi$^2^ test for categorical variables and *t*-test for continuous variables. *p* < 0.05

### Comparison of serum Cu and Zn levels and SCZR between AML patients and healthy controls

When comparing the serum Cu and Zn levels and SCZR of the AML patient group with those of the healthy control group, the results showed that the SCL and SCZR of patients in the AML group were higher than those in the healthy control group. While the serum Zn levels in the AML group were lower than those in the healthy control group, the differences were statistically significant (see Table 1). The median Cu and Zn levels of the cases and controls were 1.20 mg/L and 1.01 mg/L (*P* < 0.001) and 0.68 mg/L and 0.86 mg/L (*P* < 0.001), respectively. The SCZRs of the cases and controls were 1.74 and 1.21, respectively (*P* < 0.001). Among 105 AML patients, 23 patients showed elevated Cu levels (21.9%), 80 patients showed Zn deficiency (77.1%), and 61 patients had higher SCZR (58%).

### Associations between SCL and SCZR and clinical parameters of AML patients

The clinical, laboratory, and cytogenetic data of all AML patients are summarized in Supplementary 2 and 3. According to the SCL, the AML patients were divided into elevated and nonelevated groups. Elevated SCL was positively correlated with CRP levels (*P* = 0.001) and bone marrow blast percentages (*P* = 0.012) and was negatively correlated with ALB levels (*P* = 0.003) and *CEPBA* double mutation (*P* = 0.036). The variables with *P* < 0.1 in the above univariate analysis were included in the multivariate logistic regression analysis, and the results showed that the CRP level was closely correlated with the patient's SCL (Supplementary 4).

The AML patients were divided into a high SCZR group and a low SCZR group according to the cut-off value of the ROC curve. Compared to low SCZR, high SCZR positively correlated with CRP level (*P* = 0.004), bone marrow blast percentage (*P* = 0.046), and cytogenetic risk stratification (*P* = 0.001) and negatively correlated with haemoglobin level (*P* = 0.002), ALB level (*P* < 0.001), and PNI (*P* = 0.001). Patients with a mononuclear lineage (M4/M5) had a relatively higher SCZR (*P* = 0.001). Multivariate logistic regression analysis showed an independent association of the ALB level with SCZR (Supplementary 4).

### The impact of SCL and SCZR on the early outcomes of AML patients

To clarify the effect of SCL and SCZR on the early outcomes of AML patients, a total of 62 patients with AML who were regularly treated were enrolled in this analysis. These patients were aged 18–65 years old and received at least 2 courses of consolidation therapy. According to the critical point of SCL and SCZR, patients were divided into two groups and were compared with regard to early mortality, 1 course of complete remission rate, 2 courses of CR rate, and CTCAE classification during induction chemotherapy. The results showed that compared with the lower SCZR group, patients in the higher SCZR group had a lower rate of CR (*P* = 0.014) and a higher early mortality tendency (*P* = 0.057) after one course of induction chemotherapy. Although the latter showed nonsignificant differences, all 5 patients who died early were in the high SCZR group. Elevated Cu levels were correlated with a higher level of CTCAE classification (*P* = 0.002) (Table 2).

**Table 2:** The impact of serum copper and SCZR on the early outcomes in AML patients (*N* = 62)

Variable	ALL(n = 62)	Serum copper				SCZR			
		**Non-Elevated** **(*n* = 52)**	**Elevated** **(*n* = 10)**	**t/**$\upchi$^**2**^	**p-value**	**Low** **(*n* = 28)**	**High** **(*n* = 34)**	**t/**$\upchi$^**2**^	***p*-value**
**Early death**				2.291	0.180*			4.479	0.058
Y	5(8.1)	3 (5.8)	2 (20.0)			0(0)	5(14.7)		
N	57(91.9)	49 (94.2)	8 (80.0)			28(100)	29(85.3)		
**CR1**				1.598	0.206			6.014	0.014
Y	36 (58.1)	32 (61.5)	4 (40.0)			21(75.0)	15(44.1)		
N	26 (41.9)	20 (38.5)	6 (60.0)			7(25.0)	19(55.9)		
**CR2**				0.200	0.722			3.529	0.060
Y	41 (66.1)	35 (67.3)	6 (60.0)			22(78.6)	19 (55.9)		
N	21 (33.9)	17 (32.7)	4 (40.0)			6 (21.4)	15 (44.1)		
**CTCAE**				9.198	0.002			2.087	0.200
≤ 2	27 (43.5)	27 (51.9)	0 (0)			15 (53.6)	12 (35.3)		
> 2	35 (56.5)	25 (48.1)	10 (100)			13 (46.4)	22 (64.7)		

### The impact of SCL and SCZR on the DFS of patients with de novo AML

**Fig. 2:**
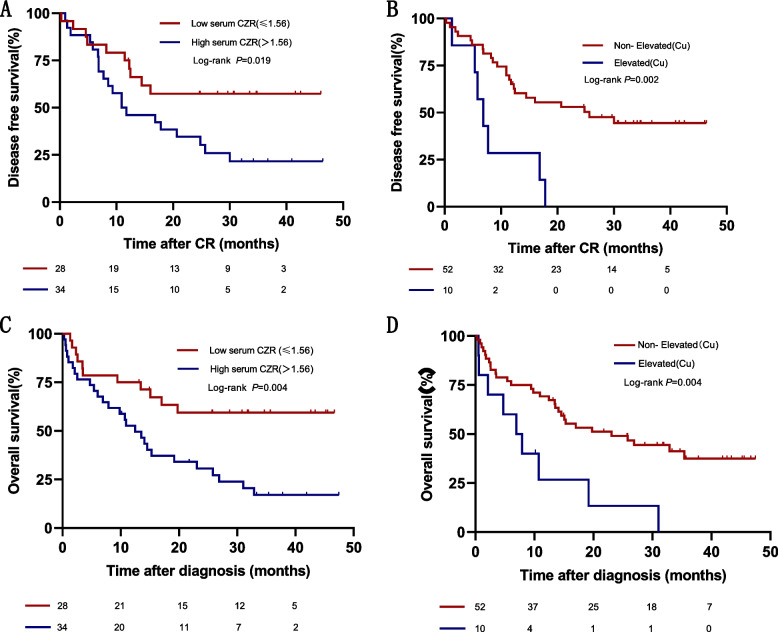
Kaplan–Meier plots for disease-free survival and SCZR (**A**) and serum copper level (**B**) in AML patients. Kaplan–Meier plots for overall survival and SCZR (**C**) and serum copper level (**D**) in AML patients

The Kaplan–Meier plot suggested that AML patients with a lower SCZR had better DFS (log-rank test, *P* = 0.019) than those with a relatively higher SCZR (Fig. 2A). Similarly, AML patients with a lower SCL were found to have a better DFS than those with elevated levels (log-rank test, *P* = 0.002; Fig. 2B). In the univariate Cox regression analyses, age, cytogenetic risk stratification, SCL, and SCZR were significantly associated with DFS (see Table 3). In the multivariate analysis, we found that in addition to cytogenetic risk stratification, SCL was independently associated with the DFS of AML patients. The multivariate-adjusted hazard ratio (HR) was 2.548 (95% CI = 1.193–5.443; *P* = 0.016) (see Table 4).

**Table 3:** The impact of serum copper and SCZR on DFS and OS of AML patients (*N* = 62)

Variable	DFS		OS	
	**HR(95%Cl)**	***p*-value**	**HR(95%Cl)**	***p*-value**
**Sex** **(F vs. M)**	0.708(0.385–1.300)	0.265	0.996(0.520–1.910)	0.991
**Age(years)** **(≥ 50vs. < 50)**	1.712(0.917–3.196)	0.091	2.202(1.150–4.215)	0.017
**BMI** (kg/m2)				
Underweight/ Normal (< 25)	Reference		Reference	
Overweight/Obesity (≥ 25)	1.481 (0.778–2.817)	0.232	1.047(0.519–2.112)	0.898
**WBC(× 10**^**9**^**/L)** (≥ 100vs. < 100)	1.990(0.858–4.612)	0.109	2.526(1.066–5.987)	0.035
**HGB(g/L)** (< 80vs. ≥ 80)	0.787(0.426–1.456)	0.446	0.945(0.492–1.815)	0.864
**PLT(× 10**^**9**^**/L)** (< 100vs. ≥ 100)	0.812(0.361–1.830)	0.616	0.695(0.305–1.580)	0.385
**BM blast percentage(%)** (≥ 60vs. < 60)	1.379(0.713–2.601)	0.321	1.291 (0.665–2.505)	0.450
**ALB(g/L)**		0.056		0.053
< 35	Reference		Reference	
35–39	1.265(0.650–2.463)	0.489	1.171(0.612–2.240)	0.658
> 39	0.334(0.112–0.993)	0.049	0.238(0.070–0.810)	0.030
**FAB classification**		0.387		0.425
M0	Reference		Reference	
M1	1.091(0.292–4.079)	0.897	0.886(0.211–3.728)	0.869
M2	0.508(0.186–1.384)	0.185	0.496(0.179–1.373)	0.177
M4/5	0.707(0.258–1.936)	0.499	0.801(0.290–2.211)	0.668
**ELN risk groups**		0.019		0.007
Favorable	Reference		Reference	
Intermediate	2.462(1.162–5.217)	0.019	3.102(1.404–6.854)	0.005
Adverse	2.560(1.223–5.359)	0.013	3.006(1.362–6.634)	0.006
**Serum copper**				
Non- Elevated	Reference		Reference	
Elevated	2.839(1.361–5.922)	0.005	2.933(1.362–6.316)	0.006
**Serum zinc**				
Non- Reduced	Reference		Reference	
Reduced	1.676(0.775–3.623)	0.189	2.151(0.899–5.148)	0.085
**SCZR**				
≤ 1.56	Reference		Reference	
> 1.56	2.084(1.094–3.971)	0.026	2.701(1.336–5.462)	0.006

**Table 4:** Multivariate analysis of the associations between SCZR, serum copper and prognosis in AML patient(*N* = 62)

Variable	DFS		OS	
	**HR(95%Cl)**	***p*-value**	**HR(95%Cl)**	***p*-value**
**SCZR be involved in model**				
**Risk groups**		0.044		0.029
Favorable	Reference		Reference	
Intermediate	2.245(1.048–4.809)	0.037	2.523(1.108–5.743)	0.027
Adverse	2.333(1.109–4.910)	0.026	2.683(1.210–5.953)	0.015
**Copper/zinc ratio**				
≤ 1.56	Reference		Reference	
> 1.56	1.834(0.955–3.525)	0.069	2.221(1.077–4.580)	0.031
**Serum copper be involved in model**				
**Risk groups**		0.036		0.016
Favorable	Reference		Reference	
Intermediate	2.474(1.164–5.258)	0.019	3.045(1.356–6.838)	0.007
Adverse	2.202(1.034–4.689)	0.041	2.505(1.110–5.655)	.0270
**Serum copper**				
Non-Elevated	Reference		Reference	
Elevated	2.548(1.193–5.443)	0.016	2.674(1.189–6.013)	0.017

### The impact of SCL and SCZR on the overall survival of patients with de novo AML

The Kaplan–Meier plot suggested that AML patients with a lower SCL were associated with better OS than those with a lower SCL (log-rank test, *P* = 0.004; Fig. 2C) (median survival was 26.25 months vs. 10.4 months). Meanwhile, AML patients with lower SCZR had a better OS than those with higher levels (log-rank test, *P* = 0.004; Fig. 2D) (median survival was 31.14 months vs. 17.75 months). In the univariate Cox regression analysis, older age (*P* = 0.017), increasing leukocyte count (*P* = 0.035), adverse genetic risk stratification (*P* = 0.005), higher SCL (*P* = 0.006), and higher SCZR (*P* = 0.006) were significantly associated with the OS of the patients (Table 3). In the multivariate analyses, in addition to cytogenetic risk stratification, both higher SCZR and higher SCL were associated with the OS of AML patients. The HRs were 2.465 (95% CI = 1.173–5.179; *P* = 0.017) and 2.505 (95% CI = 1.110–5.655; *P* = 0.027), suggesting that the SCL and SCZR were closely associated with the OS of AML patients (Table 4).

## Discussion

To our knowledge, this is the first and largest study to date that has investigated the influence of SCL and SCZR at diagnosis on survival in specific patients with AML. Patients with acute myeloid leukaemia are also in an acute phase at the time of initial diagnosis. A common feature of these acute conditions is an increase in the Cu to Zn ratio (CZr) [18]. Hence, in addition to measuring the serum copper and zinc concentrations themselves, paying attention to the ratio between Cu and Zn may have its special significance. Some studies have also revealed that these dysregulation of CZR may have clinical significance, as a prognostic and/or predictive biomarker of treatment response in cancers, and its clinical application may guide the selection of optimal treatment strategies in the future [19]. In haematological malignancies, one study indicated that elevated serum Cu levels were associated with either relapse or disease progression, whereas normal Cu levels were linked with remission or stable disease. Furthermore, they found an association between high SCL and several adverse prognostic markers in CLL [20], which was later confirmed by another study and indicated that serum Cu could be a valuable prognostic marker in B-cell chronic lymphocytic leukemia [9]. However, the association of SCL and SCZR with the survival of AML patients is unclear. The outcome of AML is heterogeneous, with both patient-related and disease-related factors contributing to an individual patient’s long-term survival. Accurate prognostic assessment of AML is imperative but not fixed. A recent study showed the impact of a multimetal score on AML patient survival, which indicated that these metals might indicate prognostic value when applied to a specific population of front-line AML patients [21]. In our study, in addition to cytogenetic stratification, serum Cu levels were associated with DFS and OS, and SCZR was only associated with OS in these younger de novo adult AML patients. Moreover, SCZR was associated with the ELN risk group and CR1, and the association indicated the prognostic value of these markers in newly diagnosed AML patients.

Essential elements, Cu and Zn, are involved in oxidative stress in cells, and the alterations of these trace elements are significant and can play a role in the occurrence, development, and even prognosis of cancers [8, 9, 11, 12, 22]. Studies from different countries have shown the levels of these trace elements with regional characteristics. In contrast to our results, data from MD Anderson in the United States suggest that serum zinc levels are not significantly reduced in patients with acute myeloid leukaemia [21]. However, most investigations reported an increased Cu concentration and a decreased Zn concentration in AML patients, and a meta-analysis analysed the data and confirmed this phenomenon [11, 23, 24]. However, no data were available about the serum Cu and Zn status in patients with AML from Northeast China. In this study, we enrolled 105 newly diagnosed adult AML patients and first confirmed the results in these AML patients from our region. In 82 controls, the median serum Zn level was 0.861 mg/L, similar to recent results from our centre [25], but was significantly lower than the median levels reported in other studies in China, such as among healthy residents of Jinan, China (Zn level, 1.32 ± 0.49 mg/L) [26]. Compared to healthy controls, the serum levels of Zn in AML patients were significantly decreased, while the serum levels of Cu were significantly increased (Table 1). Meanwhile, the SCZR showed a significant difference between AML patients and healthy controls. However, the clinical significance of these changes is not clear.

In this study, serum Cu levels were positively related to the blast percentage of bone marrow and negatively related to *CEBPAdm*. Deficiency or overload of Cu is associated with various human diseases. Excessive free Cu can disturb Zn homeostasis, which may compromise the antioxidant defence mechanism, thereby increasing oxidative stress [27]. In this study, the inflammatory parameter CRP, which is also known as a positive acute phase protein, was mostly related to the serum Cu level. This, in turn, indicated that the increase in Cu levels might be the result of inflammatory conditions in AML patients. Ceruloplasmin (Cp), a six-copper-containing protein synthesized and secreted mainly by the liver, is a major Cu carrier in the plasma. It has been shown that Cp may increase during the acute phase reaction together with other acute phase proteins following tissue trauma or acute infection. IL-6, a proinflammatory cytokine, seems to be specifically involved in the synthesis of Cp through the transcription factor FOXO1 [28]. In this study, CRP, whose production is stimulated by IL-6 and closely related to the Cu level, indirectly supported this evidence. Moreover, in AML, a gene encoding Cp was upregulated [29]. These data might explain why the high Cu level was the result of the high expression of Cp in patients with AML. In addition to CRP, a high level of Cu was related to a lower ALB level and a high blast percentage in bone marrow but was neither related to cytogenetic risk stratification nor did it affect the CR rate in AML patients. These results indicated that the adverse impact of Cu on the clinical outcomes of AML patients was due to the imbalance of homeostasis itself and might be related to its important role in angiogenesis and leukaemia cell growth [30].

In this study, serum Zn levels were lower among 75.8% of the AML patients. The serum concentrations of zinc and copper appear to be less sensitive to nutritional changes except during periods of severe deficiency or supplementation [31].This indicated that most AML patients were under a severe condition of Zn deficiency in the circulation, although this was not associated with the clinical outcomes of AML patients. Zinc is essential for a variety of enzymes and transcription factors that regulate DNA damage repair, DNA replication, cell apoptosis, and response to oxidative stress, and may upregulate the expression of tumor suppressor proteins such as p53, and zinc deficiency is also associated with immune dysfunction [32, 33]. In a randomized, controlled study of children and adolescents receiving chemotherapy for leaukemia, zinc supplementation reduced the number of infectious bouts and positively affected children's nutritional status and weight gain [34]. In another study, administration of 150 mg of zinc gluconate daily facilitated a reduction in the severity and duration of oropharyngeal mucositis in leukaemia patients [35]. The functions of the thymus and circulating T cells are highly sensitive to chemoradiotherapy, and Zn plays an important role in thymic function and immune homeostasis. A study of the role of zinc as a chemoprotectant found that after autologous stem cell transplantation high-dose oral zinc resulted in increased T-cell receptor excision rings and CD4 + naive lymphocytes and prevented torque tenovirus reactivation [36]. Zn possesses antioxidant and anti-inflammatory properties. The antioxidant properties of zinc are manifested in increased activation of antioxidant proteins and enzymes, such as glutathione and catalase. Studies have shown that zinc supplementation has an inhibitory effect on immune activation, and zinc deficiency further aggravates the activation of the NF-κB system in the case of severe infection. In vitro studies have shown that zinc reduces the activation of NF-κB and its target genes, such as TNF-α and IL-1β, and increases the gene expression of A20 and PPAR-α, two zinc finger proteins with anti-inflammatory properties [37].

Compared to single trace elements, the positive value of SCZR was meaningful, and the ratio suggests that patients were in a condition of oxidative stress [27]. While oxidative stress plays an important role in the occurrence and development of leukaemia and is closely related to the treatment and prognosis of leukaemia [38]. Meanwhile, the SCZR was an indicator of the nutritional status of Zn [39]. Zn deficiency should be highly suspected in individuals with high SCZR. It is assumed that 75%–85% (9–14 μmol/L) of Zn in the body is bound to albumin, which is the major part of the Zn exchange pool. In this study, SCZR was closely related to ALB and CRP levels, which were reported to be associated with adverse outcomes in patients with newly diagnosed AML undergoing intensive induction [40, 41]. Previous studies revealed the validity of the SCZR for the severity of nutritional status, inflammation, oxidative stress, immune dysfunction, and infection associated with Zn deficiency [42]. One study suggested that an increased SCZR, especially serum Cu concentration, was associated with an increased risk of incident infections in middle-aged and older men in Eastern Finland [43]. In our study, we observed a higher incidence of nonrelapse death, where infection was one of the crucial causes. Furthermore, we observed that Cu levels were associated with infection severity during induction chemotherapy and the association of SCZR with PNI, which was related to the nutrition status of the AML patients. This indicated that SCL and SCZR could serve as biomarkers in the general evaluation of nutrition status, oxidative stress, and stability of the internal environment. Meanwhile, SCZR was positively related to ELN risk stratification, and bone marrow blast percentage was negatively related to *CEBPA dm*. These results indicated that SCL and SCZR could present both patient-related factors and leukaemia-related factors that were associated with the prognosis of AML. Therefore, they may serve as important predictive biomarkers for the clinical outcomes of AML patients undergoing intensive chemotherapy.

The strengths of our study include the prospective case-cohort study with newly diagnosed AML patients, and all patients received standardized 3 + 7 induction chemotherapy and high-dose cytarabine-based consolidation treatment. In addition, we used inductively coupled plasma spectrometry methods for the measurement of serum element levels, which is considered to be the most reliable method for trace element determination. Moreover, the current first-determined associations of SCL and SCZR with clinical outcomes of AML may provide more information about the biological effect of these trace elements on AML prognosis; at the same time, regulating and targeting the homeostasis of trace elements may have important therapeutic implications [19].

There were several limitations in the current study. First, the sample size of the patients was relatively small. This may lead to limited power for multivariable modelling in assessing the independent prognostic values of cytogenetics, SCL, and SCZR simultaneously. The prognostic values of SCL and SCZR and DFS or OS need to be confirmed with more studies with larger sample sizes in prospective clinical trials. Second, this is a hospital case–control cohort study, and there may exist some choice bias with the population enrolled. Randomized controlled trial studies are needed to determine whether regulation of these trace elements can improve the prognosis of AML patients.

## Conclusions

The current study demonstrated that SCL and SCZR were significantly associated with the survival of AML patients, and both may serve as biomarkers in predicting the prognosis of AML patients. More studies are required to determine whether mineral regulation can improve the prognosis of AML patients and to analyse the roles of Zn and Cu in the progression of AML.

## Supplementary Information

**Additional file 1.**

## Data Availability

All data generated or analysed during this study are included in this published article and its supplementary information files.

## Abbreviations

AML: Acute myeloid leukaemia
CR: Complete remission
OS: Overall survival
TRM: Treatment- related mortality
Zn: Zinc
Cu: Copper
SCL: Serum copper level
SCZR: Serum copper to zinc ratio
WHO: World Health Organization
CLAG: Cladribine, cytarabine, G-CSF
PR: Partial remission
CTCAE: Common Terminology Criteria for Adverse Events
ICP–MS: Inductively coupled plasma spectrometry
ALB: Albumin
CRP: C-reactive protein
PNI: Prognostic nutritional index
ROC: Receiver operating characteristic
AUC: Area under the curve
CLL: Chronic lymphocytic leukaemia
Cp: Ceruloplasmin
