# Supplementary material for: The trace that is valuable: serum copper and copper to zinc ratio for survival prediction in younger patients with newly diagnosed acute myeloid leukaemia
Source: BMC Cancer. 2023 Jan 5;23:14. doi: 10.1186/s12885-022-10486-7 (PMC9817254; doi:10.1186/s12885-022-10486-7)
Supplement: Supplementary file 1 — Additional file 1. [file 12885_2022_10486_MOESM1_ESM.docx]

**Supplementary1. Patient characteristics and serum copper concentration in patients with AML(N=62)**

| Characteristics |  | **AML** |
| --- | --- | --- |
| **Age（years）,median(range)** |  | 46(19,65) |
| **Gender** |  |  |
| Male |  | 24(38.7%) |
| Female |  | 38 (61.3%) |
| **Serum copper（mg/L）,median(range)** |  | 1.193(0.437) |
| Low |  | 1(1.6%) |
| Normal |  | 51(82.3%) |
| High |  | 10(16.1%) |
| **Serum zinc（mg/L） median(range)** |  | 0.670(0.180) |
| Low |  | 47(75.8%) |
| Normal |  | 17(24.2%) |
| High |  | 0(0%) |
| **copper/zinc ratio** |  | 1.682(0.901) |
| Low |  | 28(45.2%) |
| High |  | 34(54.8%) |
| **Risk stratification** |  |  |
| Favorable |  | 29 (46.8%) |
| Intermediate |  | 16(25.8%) |
| Adverse |  | 17(27.4%) |
| **Death** |  | 38(61.3%) |
| Non-relapse mortality  Relapse mortality  Early death |  | 29(76.3%)  4  5（8.1%） |
| **Treatment** |  |  |
| HSCT |  | 7(10.4%) |

Supplementary 2. Demographic and clinical correlates of serum copper and copper-zinc ratio in AML patients

| **Variable** |  | **All patients**  **（n=105）** | **serum copper** | | | |  | **SCZR** | | | |
| --- | --- | --- | --- | --- | --- | --- | --- | --- | --- | --- | --- |
|  |  |  | **Non- Elevated（N=82）** | **Elevated（N=23）** | **t/χ²/U** | **p-value** |  | **Low**  **(N=44)** | **High**  **(N=61)** | **t/χ²/U** | **p-value** |
| **Sex(%)** |  |  |  |  | 1.783 | 0.182 |  |  |  | 1.082 | 0.298 |
| M |  | 51(48.6) | 37(45.1) | 14(60.9) |  |  |  | 24(54.5) | 27(44.3) |  |  |
| F |  | 54(51.4) | 45(54.9) | 9(39.1) |  |  |  | 20(45.5) | 34(55.7) |  |  |
| **Age(years)** |  | 50.0(38.5-57.5) | 49.0(37.0-56.3) | 51.0(45.0-59.0) | 843.00 | 0.438 |  | 46.0(35.3-54.8） | 51.0(45.0-59.0） | 1079.0 | 0.087 |
| **BMI（kg/m^2^）** |  | 23.34(20.45-25.31) | 23.29(20.40-25.22) | 23.46(20.63-25.39) | 883.50 | 0.645 |  | 24.01(21.06-25.87) | 22.49(19.95-25.03) | 1102.0 | 0.119 |
| **WBC（×10^9^/L）** |  | 16.24(2.92-51.03) | 12.38(2.66-47.27) | 34.30(3.86-82.42) | 781.50 | 0.211 |  | 16.24(6.06- 46.88) | 13.23(2.27-61.04) | 1255.0 | 0.572 |
| **HGB（g/L）** |  | 74.82±23.72 | 77.16±22.89 | 66.48±25.26 | 1.933 | 0.056 |  | 82.95±23.04 | 68.95±22.61 | 2.824 | 0.002 |
| **PLT（×10^9^/L）** |  | 34.00(18.00-71.50) | 34.00(18.00-68.25) | 35.00(23.00-74.00) | 829.50 | 0.379 |  | 23.00(13.75-60.00) | 35.00(23.50-75.00) | 1042.0 | 0.051 |
| **PB blast percentage** |  | 41.00(7.50-78.50) | 35.00(5.00-70.25) | 65.00(13.00-91.00) | 726.00 | 0.093 |  | 40.00(4.75-68.13) | 47.00(8.50-85.50) | 1224.0 | 0.443 |
| **BM blast percentage** |  | 63.00(44.50-85.00) | 61.25(42.00-81.13) | 84.50(52.00-92.50) | 617.00 | 0.012 |  | 60.75(33.63-78.38) | 70.50(46.25-88.75) | 1035.0 | 0.046 |
| **ALB(g/L)(N=101)** |  | 35.72±4.24 | 36.20±4.21 | 34.00±3.96 | 2.196 | 0.030 |  | 37.74±3.74 | 34.22±3.97 | 4.506 | ＜0.001 |
| **TG（mmol/L）(N=75)** |  | 1.08(0.75-1.53) | 1.10(0.76-1.63) | 1.02(0.65-1.34) | 408.50 | 0.285 |  | 1.10(0.79-1.63) | 1.06(0.67-1.38) | 601.0 | 0.307 |
| **TC（mmol/L）(N=75)** |  | 2.99±0.72 | 3.04±0.71 | 2.83±0.78 | 1.052 | 0.296 |  | 3.04±0.73 | 2.95±0.73 | 0.540 | 0.591 |
| **HDL(mmol/L)(N=75)** |  | 0.81(0.68-0.99) | 0.80(0.69-0.97) | 0.83(0.64-1.13) | 469.0 | 0.761 |  | 0.80(0.69-0.98) | 0.83(0.66-1.07) | 649.5 | 0.613 |
| **LDL（mmol/L）(N=75)** |  | 1.72±0.53 | 1.76±0.54 | 1.59±0.50 | 1.139 | 0.258 |  | 1.75±0.59 | 1.69±0.48 | 0.527 | 0.600 |
| **CRP(mg/L)(N=53)** |  | 41.12(10.08-67.69) | 22.06(6.12-50.85) | 68.78(49.50-229.32) | 93.00 | 0.001 |  | 11.66(3.21-42.99) | 49.20(18.90-80.90) | 162.50 | 0.004 |
| **FAB classification(%)** |  |  |  |  | 3.212 | 0.276 |  |  |  | 14.575 | 0.001 |
| M0 |  | 8(7.6) | 5(6.1) | 3(13.0) |  |  |  | 0(0.0) | 8(13.1) |  |  |
| M1 |  | 6(5.7) | 4(4.9) | 2(8.7) |  |  |  | 2(4.5) | 4(6.6) |  |  |
| M2 |  | 42(40.0) | 36(43.9) | 6(26.1) |  |  |  | 26(59.1) | 16(26.2) |  |  |
| M4/5 |  | 49(46.7) | 37(45.1) | 12(52.2) |  |  |  | 16(36.4) | 33(54.1) |  |  |
| **ELN risk goups (%)** |  |  |  |  | 2.788 | 0.248 |  |  |  | 12.650 | 0.002 |
| Favorable |  | 38(36.2) | 33(40.2) | 5(21.7) |  |  |  | 24(54.5) | 14(23.0) |  |  |
| Intermediate |  | 32(30.5) | 24(29.3) | 8(34.8) |  |  |  | 7(15.9) | 25(41.0) |  |  |
| Adverse |  | 35(33.3) | 25(30.5) | 10(43.5) |  |  |  | 13(29.5) | 22(36.1) |  |  |
| **PNI(N=100)** |  | 46.03(41.26-56.57) | 46.58(45.31-57.24) | 45.09(40.98-55.00) | 837.00 | 0.861 |  | 53.12(43.89-61.38) | 43.30(40.78-50.09) | 753.0 | 0.001 |

Supplementary 3. Correlation between levels of serum copper and SCZR and molecular genetic features

| **Variable** |  | **All patients**  **（n=96）** | **serum copper** | | | |  | **SCZR** | | | |
| --- | --- | --- | --- | --- | --- | --- | --- | --- | --- | --- | --- |
|  |  |  | **Non- Elevated（N=75）** | **Elevated（N=21）** | **t/χ²/U** | **p-value** |  | **Low**  **(N=40)** | **High**  **(N=56)** | **t/χ²/U** | **p-value** |
| ***CEBPA*** |  |  |  |  | 4.978 | 0.036* |  |  |  | 7.335 | 0.010* |
| Wild type |  | 81(84.4) | 60（80.0） | 21（100） |  |  |  | 29（75.0） | 52（92.9） |  |  |
| Mutated |  | 15(15.6) | 15（20.0） | 0（0） |  |  |  | 11（15.0） | 4（6.6） |  |  |
| ***NPM1*** |  |  |  |  | 0.487 | 0.485 |  |  |  | 6.476 | 0.014* |
| Wild type |  | 74(77.1) | 59（78.7） | 15(71.4) |  |  |  | 36（90.0） | 38（67.9） |  |  |
| Mutated |  | 22(22.9) | 16（21.3） | 6(28.6） |  |  |  | 4（10.0） | 18（32.1） |  |  |
| ***FLT3*** |  |  |  |  | 1.037 | 0.308 |  |  |  | 2.789 | 0.095 |
| Wild type |  | 68(70.8) | 55(73.3) | 13(61.9) |  |  |  | 32(80.0) | 36(64.3) |  |  |
| Mutated |  | 28(29.2) | 20(26.7) | 8(38.1) |  |  |  | 8(20.0) | 20(35.7) |  |  |
| ***DNMT3A*** |  |  |  |  |  |  |  |  |  | 0.992 | 0.319 |
| Wild type |  | 77(80.2) | 63(84.0) | 14(66.7) | 3.105 | 0.078 |  | 34(85.0) | 43(76.8) |  |  |
| Mutated |  | 19(19.8) | 12(16.0) | 7(33.3) |  |  |  | 6(15.0) | 13(23.2) |  |  |
| ***ASXL1*** |  |  |  |  | 0.552 | 0.728 |  |  |  | 3.450 | 0.063 |
| Wild type |  | 82(85.4) | 63(84.0) | 19(90.5) |  |  |  | 31(77.5) | 51(91.1) |  |  |
| Mutated |  | 14(14.6) | 12(16.0) | 2(9.5) |  |  |  | 9(22.5) | 5(8.9) |  |  |
| ***RUNX1*** |  |  |  |  | 0.023 | 1.000 |  |  |  | 0.013 | 1.000 |
| Wild type |  | 86(89.6) | 67(89.3) | 19(90.5) |  |  |  | 36(90.0) | 50(89.3) |  |  |
| Mutated |  | 10(10.4) | 8(10.7) | 2(9.5) |  |  |  | 4(10.0) | 6(10.7) |  |  |
| ***TP53*** |  |  |  |  | 0.102 | 1.000 |  |  |  | 0.183 | 0.691 |
| Wild type |  | 90(93.8) | 70(93.3) | 20(95.2) |  |  |  | 37 (92.5) | 53(94.6) |  |  |
| Mutated |  | 6(6.2) | 5(6.7) | 1(4.8) |  |  |  | 3(7.5) | 3(5.4) |  |  |
| ***TET2*** |  |  |  |  | 3.840 | 0.063 |  |  |  | 0.392 | 0.531 |
| Wild type |  | 84(87.5) | 63(84.0) | 21(100) |  |  |  | 34(85.0) | 50(99.3) |  |  |
| Mutated |  | 12(12.5) | 12(16.0) | 0(0) |  |  |  | 6(15.0) | 6(10.7) |  |  |
| ***IDH1*** |  |  |  |  | 7.513 | 0.020 |  |  |  | 1.646 | 0.396 |
| Wild type |  | 90(93.8) | 73(97.3) | 17(81.0) |  |  |  | 39(97.5) | 51(91.1) |  |  |
| Mutated |  | 6(6.2) | 2(2.7) | 4(19.0) |  |  |  | 1(2.5) | 5(8.9) |  |  |
| ***IDH2*** |  |  |  |  | 0.254 | 1.000 |  |  |  | 0.744 | 0.446 |
| Wild type |  | 89(92.7) | 69(92.0) | 20(95.2) |  |  |  | 36(90.0) | 53(94.6) |  |  |
| Mutated |  | 7(7.3) | 6(8.0) | 1(4.8) |  |  |  | 4(10.0) | 3(5.4) |  |  |
| **Gene mutations** |  |  |  |  | 0.945 | 0.391 |  |  |  | 0.058 | 1.000 |
| No mutation |  | 2(2.1) | 1(1.3) | 1(4.8) |  |  |  | 1(2.5) | 1(1.8) |  |  |
| Mutations |  | 94(97.9) | 74(98.7) | 20(95.2) |  |  |  | 39(97.5) | 55(98.2) |  |  |
| **Gene mutations** |  |  |  |  | 0.239 | 0.734 |  |  |  | 0.020 | 0.887 |
| ≥2 |  | 81(84.4) | 64(85.3) | 17(81.0) |  |  |  | 34(85.0) | 47(83.9) |  |  |
| ＜2 |  | 15(15.6) | 11(14.7) | 4(19.0) |  |  |  | 6(15.0) | 9(16.1) |  |  |
| **Gene mutations** |  |  |  |  | 0.120 | 0.729 |  |  |  | 0.244 | 0.621 |
| ≥3 |  | 58(60.4) | 46(61.3) | 12(57.1) |  |  |  | 23(57.5) | 35(62.5) |  |  |
| ＜3 |  | 38(39.6) | 29(38.7) | 9(42.9) |  |  |  | 17(42.5) | 21(37.5) |  |  |

**Supplementary 4 Multivariate analysis of serum copper and copper/zinc ratio in AML patients(N=105)**

| **Variable** | **Serum copper** | | |  | **SCZR** | | |
| --- | --- | --- | --- | --- | --- | --- | --- |
|  | **OR** | **95%CI** | **p-value** |  | **OR** | **95%CI** | **p-value** |
| CRP | 1.015 | 1.004-1.027 | 0.009 |  | 1.017 | 0.999-1.035 | 0.053 |
| ALB | 0.880 | 0.715-1.084 | 0.229 |  | 0.736 | 0.609-0.889 | 0.001 |
